# Mitigating Suicide Risk During the Military-to-Civilian Transition: The VA Veteran Sponsorship Initiative

**DOI:** 10.3390/ijerph23040519

**Published:** 2026-04-17

**Authors:** Joseph C. Geraci, David E. Goodrich, Erin P. Finley, Amanda L. Reed, Michael Eastman, Danielle Bracco, A. Solomon Kurz, Emily R. Edwards, Christine Eickhoff, Chien J. Chen, Andrea MacCarthy, Brian Roeder, Chris Paine, Alberto Feliciano, Brigid Connelly, Eric Andrew Nelson, Sarah Rachael Karkout, Nicholas Ahari, Nicholas R. Lindner, Jack Besser, Megan McFadyen-Mungall, Madeleine Allen, Samantha Gitlin, Matthew R. Augustine, Travis Bellotte, Leah Smith, Smita Badhey, Balavenkatesh Kanna, Brian Westlake, Meenakshi Zaidi, Rakeshwar S. Guleria, Brian P. Marx, Nicolle Marinec, Jason Wesbrock, Andy Cox, Kevin D. Admiral, Richard W. Seim, Ronald C. Kessler, Marianne Goodman

**Affiliations:** 1 VA Veterans Integrated Service Network (VISN) 2 Mental Illness Research, Education and Clinical Center, James J. Peters VA Medical Center, Bronx, New York, NY 10468, USA; 2 VISN 17 Center of Excellence for Research on Returning War Veterans, Doris Miller VA Medical Center, Central Texas Veterans Health Care System, Waco, TX 76711, USA; 3 Teachers College, Columbia University, New York, NY 10027, USA; 4 Department of Psychiatry, Icahn School of Medicine at Mount Sinai, New York, NY 10029, USA; 5 Center for Health Equity Research and Promotion, VA Pittsburgh Healthcare System, Pittsburgh, PA 15240, USA; 6 Department of General Medicine, University of Pittsburgh School of Medicine, Pittsburgh, PA 15213, USA; 7 Center for the Study of Healthcare Innovation, Implementation, and Policy (CSHIIP), VA Greater Los Angeles Healthcare System, Los Angeles, CA 90073, USA; 8 James J. Peters VA Medical Center, Bronx, New York, NY 10468, USA; 9 Onward Ops, Washington, DC 20037, USA; 10 VA National Center for Healthcare Advancement and Partnership, Washington, DC 20420, USA; 11 Center for the Study of Traumatic Stress, Department of Psychiatry, Uniformed Services University of the Health Sciences, Bethesda, MD 20814, USA; 12 Denver Center of Innovation for Veteran-Centered and Value-Driven Care, VA Eastern Colorado Health Care System, Aurora, CO 80045, USA; 13 VA VISN 2 Clinical Resource Hub, Tarrytown, NY 10591, USA; 14 National Center for PTSD, VA Boston Healthcare System, Boston, MA 02130, USA; 15 Department of Psychiatry, Boston University Aram V. Chobanian & Edward Avedisian School of Medicine, Boston, MA 02118, USA; 16 Center for Clinical Management Research/Health Systems Research, LTC Charles S. Kettles, VA Medical Center, Ann Arbor Healthcare System, Ann Arbor, MI 48105, USA; 17 III Armored Corps, U.S. Army, Fort Hood, Killeen, TX 76544, USA; 18 Department of Health Care Policy, Harvard Medical School, Boston, MA 02115, USA

**Keywords:** transitioning servicemembers/veterans, adaptations, deadly gap, suicide prevention, peer support

## Abstract

**Highlights:**

**Public health relevance—How does this work relate to a public health issue?**

- The youngest veterans in the United States (U.S.) are experiencing a suicide epidemic as the suicide rates for veterans aged 18 to 34 are higher than any other age group and more than doubled from 2001 and 2022.
- Suicide risk is especially elevated during the transition from active-duty military service to civilian life- the “deadly gap”, as suicide rates for servicemembers exiting military service are nearly three times higher during the first year post-military discharge.

**Public health significance—Why is this work of significance to public health?**

- Many national calls to action and congressional legislative actions have directed the Departments of War (DoW) and Veterans Affairs (VA) to address the problem of high suicide risk among servicemembers transitioning to civilian life.
- The VA developed a preventative, public health approach to the problem that prioritizes transitioning servicemembers receiving VA clinical services and support from community-based organizations that targets social determinants of health (e.g., poor social connectedness, financial concerns, relationship distress).

**Public health implications—What are the key implications or messages for practitioners, policy makers and/or researchers in public health?**

- VA Veteran Sponsorship Initiative (VSI) is a public-private-partnership between federal and community partners that aims to decrease suicides by providing a VA-certified volunteer peer-sponsor, managed by Onward Ops; connection to community services and seamless access to VA healthcare through the VA National Virtual Care Clinic for Transitioning Veterans.
- In preparation for national implementation, the evaluators conducted a quasi-experimental, matched-cohort pilot that demonstrated the feasibility of an adapted VSI protocol and built upon the evidence-basis for VSI by identifying a statistically significant increase in VA primary care utilization and decrease in suicide attempts for VSI participants compared to non-VSI matched controls.

**Abstract:**

A suicide epidemic exists among young U.S. veterans, with risk especially elevated in the first year of transition for the 200,000 servicemembers exiting the military annually. The VA Veteran Sponsorship Initiative (VSI) is a public–private-partnership between federal and community partners that aims to decrease suicides by providing a VA-certified volunteer peer sponsor and connection to community services. Onward Ops is a key community-based national program that enrolls, matches and manages the relationship between servicemembers and sponsors. A prior randomized controlled trial showed that the effectiveness of community interventions can be enhanced when augmented by an Onward Ops sponsor. In preparation for national implementation, we conducted a quasi-experimental, matched-cohort pilot to evaluate the feasibility of an adapted VSI protocol and then assessed effectiveness. The adaptations were executed using the Framework for Reporting Adaptations and Modifications-Enhanced between April 2021 and April 2023. The formative results supported the feasibility of the adaptations to enable proactive enrollment on military installations and expand data infrastructure, partnerships, peer sponsors, and VA clinical services. We then assessed the effectiveness for outcomes not studied in the original VSI trial for active-duty soldiers who enrolled between April and December 2023. After nearest-neighbor matching, the sample included 551 VSI participants and 551 soldiers transitioning as usual. The point-probability contrast or risk differences from the conditional logistic regression model indicated that the VSI caused a statistically significant increase in VA primary care utilization of 0.198 and a statistically significant decrease in suicide attempts of −0.019, both assessed 10 months post-military discharge. The study demonstrated the utility of public–private-partnerships, peer-sponsorship programs and enhanced VA services to support servicemembers during transition.

## 1. Introduction

The youngest veterans in the United States (U.S.) are experiencing a suicide epidemic. The suicide rates for veterans aged 18 to 34 are higher than any other age group and more than doubled from 2001 to 2022, from 24 per 100,000 to 55 per 100,000 [1]. Suicide risk is especially elevated during the transition from active-duty military service to civilian life, as shown by Shen and colleagues [2], who found that suicide rates for servicemembers exiting military service were nearly three times higher during the first year post-military discharge. This “deadly gap” for transitioning servicemembers/veterans (TSMVs) applies to the approximately 200,000 servicemembers exiting active-duty military service each year [3,4,5].

### 1.1. National Focus

Many national calls to action and congressional legislative actions [6] have directed the Departments of War (DoW) and Veterans Affairs (VA) to address the problem of high suicide risk among TSMVs. The VA developed a preventative, public health approach to the problem that prioritizes TSMVs receiving VA clinical services and support from community-based organizations that target social determinants of health (e.g., poor social connectedness, financial concerns, relationship distress) [7,8,9]. Integrating such an approach may be beneficial and help connect TSMVs to VA care. This is critical, as most veterans who die by suicide are not engaged in VA healthcare. Recent data for veterans who died by suicide in 2021 found that 60% had not had any VA healthcare services within the prior two years and that over 50% never received any VA healthcare services [1]. Importantly, only one-fourth of TSMVs utilize any VA healthcare during the first year post-military discharge period of the deadly gap [10].

### 1.2. VA Veteran Sponsorship Initiative

Military culture tends to emphasize camaraderie and gaining support through peer relationships, particularly during periods of transition. As such, peer and/or mentorship-based programming may be particularly well suited for TSMVs [11]. In a meta-analysis of 112 studies across a variety of samples, mentoring was favorably associated with relational, career, and mental health outcomes [12]. Veteran-specific research also indicates the value of peer mentoring-based interventions in educational, healthcare, and criminal justice contexts [13,14,15].

The VA Veteran Sponsorship Initiative [16] is a public–private partnership of VA with the DoW, national nonprofit organizations, and community organizations. The VSI aims to reduce suicide risk for TSMVs by reducing physical/psychological pain, increasing connectedness, and reducing TSMV access to lethal means during times of acute crisis. The execution of the VSI is informed by the DoW’s Permanent Change of Station Sponsorship program [17], recommendations from the DoW’s Best Practices Identified for Peer Support Programs [18], the Three-Step Theory of Suicide [19,20,21], and VA’s preventative and public health approach to suicide prevention [9]. Please see Geraci and colleagues for more information [21].

The VSI was originally designed to assist TSMVs through two core elements: 1. Community-based Peer Sponsors and 2. Community Services. As part of the VSI, the Expiration Term of Service (ETS) Sponsorship Program [22], a non-profit social welfare organization that operates the Onward Ops community-based transition program for TSMVs, has the responsibility of managing these two core elements. After signing up for a peer sponsor on military bases, TSMVs are matched with sponsors optimally located in the TSMVs’ planned post-military discharge destination by Onward Ops based on similarities in individual demographics and professional interests. TSMVs are encouraged to have regular contact with their sponsors for at least six months post-military discharge through social media, email, and monthly video or in-person meetings. Prior to serving as sponsors, veteran and civilian volunteers undergo training and certification by VA trainers [23] in three key areas: (1) establishing positive interpersonal relationships with TSMVs; (2) supporting TSMVs in setting and achieving Specific-Measurable-Achievable-Relevant-Timely (SMART) goals to address their transition needs; and (3) suicide prevention skills informed by VA S.A.V.E. suicide prevention training [24] and lethal means safety training. Sponsors are also provided support from local community partners; the primary responsibilities of these partners include (1) recruiting volunteer sponsors and ensuring they attend VA certification training, (2) managing sponsors’ relationships with TSMVs in their local region, (3) submitting referrals to VA and other agencies to assist TSMVs in meeting their transition goals, and (4) monitoring TSMV progress.

An initial evaluation of the VSI (VSI trial #1; 2014–2018) [25] was carried out by a randomized controlled trial in which 200 TSMVs (mean time since discharge = 2.77 years) located in New York City were randomly assigned to participate in: (1) a waitlist control condition, (2) a community-based veteran service organization (VSO) only; or (3) a community-based VSO with an Onward Ops sponsor. Results showed that participating in a community-based VSO with an Onward Ops sponsor was associated with significantly fewer reintegration difficulties [26] and greater social connectedness than participating in a community-based VSO alone.

Following this research, VA funded two randomized hybrid type 2 effectiveness–implementation trials (see online Supplementary Material Table S1 for a timeline and components of VSI studies). VSI trial #2 (2022–2025) uses a stepped-wedge design with 630 active-duty TSMVs who planned on moving to one of six Texas cities post-military discharge. TSMVs were assigned either to transition as usual (TAU; *n* = 315) or to receive VSI support (including an Onward Ops sponsor and facilitated connection to VA clinical services; *n* = 315). Enrollment for TAU started in February 2022 with a phased enrollment for the VSI beginning in November 2024. VSI trial #3 (2025–2027) focuses on a phased roll-out of the VSI to select military bases with 12,000 active-duty TSMVs moving across the U.S. The TSMVs within the top 30% of predicted suicide risk, based on machine learning models using TSMV self-report information obtained prior to separation as predictors [27], will be placed within one of three arms (Arm1/TAU, *n* = 900; Arm2/VSI = Onward Ops only, *n* = 900; Arm3/VSI = Onward Ops and facilitated connection to VA clinical services, *n* = 900). The machine learning models were developed by the DoW Study to Assess Risk and Resilience in Servicemembers (STARRS) and identified that 30% of servicemembers with the highest predicted risk included 92.5% of self-reported suicide attempts post-military discharge. Enrollment into this study begins in March 2026. VSI trials #2 and #3 further assess the effectiveness of the VSI in improving TSMVs’ reintegration difficulties [26], psychological/physical pain, connectedness, VA healthcare utilization, and suicide risk, as well as the implementation of the VSI at the local and national level and associated budget impact analysis.

### 1.3. Aims of the Current Study

To support the scalability and long-term implementation potential of the VSI program, priority adaptations were made to the initial VSI program from April 2021 to April 2023, which facilitated the enrollment of participants while on active duty. There was also an expansion in data infrastructure, partnerships with community partners and peer sponsors, and an addition of VA clinical infrastructure to address the medical needs of VSI participants. The primary aim of this study was to investigate the feasibility of making these adaptations to the VSI program. The second aim was to determine the further effectiveness of the VSI after making the adaptations, specifically for outcomes not studied in VSI trial #1 but identified as high-priority targets for TSMV wellbeing—VA primary care utilization (primary outcome) and post-military discharge suicide attempts requiring VA medical care (secondary outcome). We hypothesized that VSI participants would have higher VA primary care utilization and fewer suicide attempts post-military discharge than the TSMVs who transitioned without enrolling in the VSI. The current study served as a critical bridge between the evidence-basis determined in VSI trial #1 with 200 TSMVs in one geographic location and one community partner and the future large-scale implementation of the VSI across the U.S. with thousands of TSMVs in VSI trials #2 and #3.

## 2. Materials and Methods

This was a longitudinal, quasi-experimental and matched-cohort pilot study classified by the Central Texas VA Central Texas Healthcare System as quality improvement and a non-research program evaluation. Specifically, the study compared the outcomes of TSMVs who participated in the adapted VSI program (Arm1/VSI = Onward Ops and facilitated connection to VA clinical services, *n* = 551) and a matched sample of TSMVs (Arm2/TAU, *n* = 551) who did not participate in the VSI.

### 2.1. Sample

TSMVs were considered eligible if they were at least 18 years old and completed a military transition application managed by Onward Ops on participating U.S. Army installations while serving within the active-duty military between April 2023 and December 2023. A total of 1391 TSMVs completed the military transition application during the application window (see online Supplementary Material Figure S1 for Consolidated Standards of Reporting Trials diagram). Of these, 23 TSMVs screened positive for possible acute suicidality on the Columbia-Suicide Severity Rating Scale (C-SSRS) [28], as part of the military transition application. For safety reasons, each of these TSMVs was contacted by VA social workers for further clinical assessment and offered VSI services; they were excluded from this study. Data to support the current analysis was collected in October 2024. All TSMVs were out of the military for at least ten months at the time of data collection.

There were 551 TSMVs who completed the military transition application at Fort Hood, Texas, and elected to receive VSI services (Arm1/VSI). An additional 817 TSMVs completed the military transition application at one of three participating U.S. Army installations and were considered eligible for matching; 179 of these TSMVs requested VSI services but were placed in a TAU arm in accordance with procedures identified for a separate VSI study [21], and 638 declined VSI services during their military transition application. Of these, 551 were included in the TAU arm for analysis. We focus in the current study on Arm1/VSI (*n* = 551) compared to Arm2/TAU (*n* = 551).

### 2.2. Design

#### 2.2.1. Aim 1. Evaluate the Feasibility of Adaptations

For Aim 1, evaluators used the Framework for Reporting Adaptations and Modifications-Enhanced (FRAME) [29] and associated FRAME codebook [30] to systematically categorize adaptations made to the VSI (see online Supplementary Material Table S2 for VSI FRAME). To support the scalability and long-term implementation of the VSI program, VSI partners identified four priority adaptations:

1. TSMVs apply within the military (rather than following discharge);
2. Establish robust data infrastructure;
3. Expand community partnerships and peer sponsors;
4. Increase VA clinical capacity to address TSMV needs.

**Figure 1:**
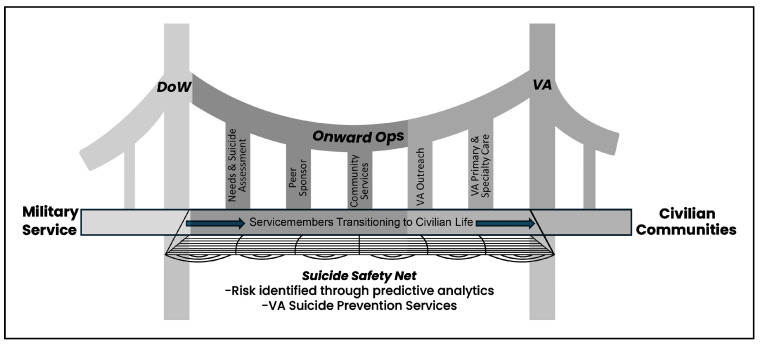
Core elements of the VA Veteran Sponsorship Initiative to bridge the “deadly gap”.

Partners believed these adaptations would allow the VSI to better serve the needs of TSMVs before, during, and immediately post-military discharge through expanding the VSI’s core elements to include 1. Needs and Suicide Assessment, 2. Community-based Peer Sponsors, 3. Community Services, 4. VA Outreach and 5. VA Primary and Specialty Care (Figure 1).

In the current study, evaluators assessed the feasibility of each adaptation to determine whether further adaptation was necessary prior to the continued evaluation of the VSI program. Toward this end, evaluators leveraged the feasibility framework developed by Gadke and colleagues [31] to identify the most appropriate dimensions of feasibility (i.e., recruitment capability, data collection procedures, integration into existing systems, practicality, acceptability), as detailed below.

##### Adaptation 1. TSMVs Apply Within the Military

Due to the design of VSI trial #1 [25], only 21% of participants enrolled while they were still within the military. Ravindran and colleagues [32] found that suicide rates after separation are time-dependent, with the highest suicide rates observed in the first year after discharge. Therefore, many researchers have recommended that the optimal time for the enrollment of TSMVs into preventive interventions is prior to military discharge [33,34,35,36]. Accordingly, in October 2022, U.S. Army leadership made the decision to have TSMVs on participating military installations complete a military transition application prior to military discharge. As part of this application, TSMVs were provided the option to select to receive an Onward Ops peer sponsor and VA services as part of their discharge process.

This adaptation aligned with the feasibility dimension of “recruitment capability.” Successful recruitment of target study participants is critical for ensuring feasibility for future trials [31]. There were two specific questions related to recruitment capability and the feasibility of this adaptation: (1) Was it feasible to recruit TSMVs prior to their military discharge? (2) Would recruited TSMVs be representative of the active-duty U.S. Army [37] with respect to rank and demographic factors?

##### Adaptation 2. Establish Robust Data Infrastructure

Future evaluation and implementation of the VSI were planned to have three primary sources of operational and clinical data not present in VSI trial #1: (1) self-report assessment data from the military transition application, (2) VA enrollment and medical records provided through the VA Corporate Data Warehouse (CDW) and (3) VA administrative and outreach data.

The military transition application includes a range of self-report assessments, including that of transition needs (e.g., employment, education, housing, food insecurity, family, medical), the Patient Health Questionnaire-2 (PHQ-2) [38], Generalized Anxiety Disorder-2 [39] the Military to Civilian Questionnaire (M2C-Q) [26], and a 17-item assessment of risk for post-military discharge suicide attempt developed with ensemble machine learning [27]. The C-SSRS [28] is included within the 17-item assessment.

Given the vast data infrastructure needed to coordinate and store VSI data, an adaptation was made to establish an initial data infrastructure to ensure that VA could receive and consolidate the aforementioned data for clinical and operational use. In April 2021, Onward Ops entered into a Cooperative Research and Development Agreement (CRADA) with the VA’s Innovation Ecosystem to establish a VA data repository to support the consolidation of TSMV data within VA. This adaptation aligned with the feasibility dimension of “data collection procedures”, which guides evaluators to consider the appropriateness of data collection procedures for the target population [31]. Our team sought to assess: (1) Was it feasible for the VA team and the evaluators to access data from each data source for every TSMV? and (2) Was it feasible for the VA team to merge all sources of data for VA clinical and operational use?

##### Adaptation 3. Expand Community Partnerships and Peer Sponsors

In VSI trial #1, there was only one local community partner, providing 97 certified sponsors. To manage projected growth, an adaptation was made by Onward Ops to expand national and local community partnerships. In April 2021, it established ten regions across the nation (see online Supplementary Material Table S3) and established partnerships with additional local community partners. Many of these partners also established partnerships with the local VA leadership within respective regions, as facilitated by the VA National Center for Healthcare Advancement and Partnerships, under its Veteran Sponsor Partnership Network [40].

This adaptation aligns with the feasibility dimension, “integration into existing systems,” given the intent to leverage existing community partners rather than creating new entities at the local level. This dimension helps to identify how well an intervention fits within the organizational structure, physical environment, or existing service delivery approach prior to initiating larger studies or expansion of interventions [31]. For the study, we evaluated: (1) What regions did TSMVs in Arm1/VSI identify most frequently as their post-military discharge destination? (2) How many community partners were within each region, with a focus on high-demand regions? (3) Are there a sufficient number of peer sponsors to meet the projected demand for sponsors in the future VSI studies?.

##### Adaptation 4. Increase VA Clinical Capacity to Address TSMV Needs

Underscoring the importance of VA healthcare, an analysis of 20 years of suicide data (2001–2021) reveals that veterans engaged in VA healthcare have shown a less sharp rise in suicide rates compared to other veterans [41]. Relatedly, the enactment of the Commander John Scott Hannon Veterans Mental Health Care Improvement Act [6] required VA to draft a strategic plan to expand healthcare coverage for TSMVs during their transition to civilian life. Within this plan, VA described its primary goal of ensuring every eligible TSMV benefits from a seamless and personalized transition process that creates a lasting and trusting veteran health experience [42]. To accomplish this goal, VA prioritized continuity of care in the plan by ensuring all TSMVs enrolled in VA care receive a timely primary care appointment after leaving active duty.

In VSI trial #1, there was no VA outreach to VSI participants nor a VA-centralized structure or process that offered access to VA primary care and other clinical services for TSMVs. Therefore, an adaptation was made to increase access for TSMVs, specifically for VSI participants. This adaptation required various complementing efforts.

First, in April 2021, Onward Ops entered into a memorandum of agreement with VA’s Undersecretary for Health that focused on coordination between the organizations and synchronization to optimize the number of TSMVs receiving VA clinical services. Second, at the same time, VA started using contact information provided by Onward Ops (with TSMV consent) to contact TSMVs via authorized VA emails and text messages while they were on active-duty. The outreach provided information to TSMVs for completing key action items related to becoming enrolled within VA (e.g., completing the VA healthcare registration/application form and applying for VA service-connected disability). VA social workers were hired and also started engaging with higher-risk TSMVs (as identified by the assessment of risk [27]) to conduct clinical suicide risk assessments and to support TSMVs in completing key action items related to enrollment and connection to VA primary care services.

Third, VA established the VA National Virtual Care Clinic for Transitioning Veterans (NVCC) in January 2022. The NVCC aimed to offer all eligible TSMVs within the VSI seamless access to VA clinical services through their nearest VA facility or through virtual services offered directly by the NVCC. Through a charter with the VA Office of Primary Care, the NVCC established four regional hubs across the nation to facilitate virtual primary care and specialty care (e.g., mental health, polytrauma assessments, and pharmacy) for TSMVs in collaboration with VA virtual care programs, such as VA Clinical Resource Hubs and facility-based telehealth services [43]. (For further information about the NVCC, including an early evaluation of program feasibility, see Kumar and colleagues [44].)

This adaptation aligned with the evaluated feasibility dimension, “practicality,” which determines whether the intervention can be used given contextual and environmental constraints such as time, resource availability, and practitioner commitment [45]. Given how critical seamless access to VA primary care is for the VSI, we evaluated: (1) What barriers exist for TSMVs to become eligible for VA clinical services? (2) What is the demand for NVCC services and most frequently diagnosed medical conditions among TSMVs in VSI? (3) Is there sufficient NVCC capability to meet the projected demand for NVCC services in the future VSI studies? (4) Are there notable sex differences in rates of medical conditions diagnosed by NVCC virtual primary care providers?

#### 2.2.2. Aim 2. Effectiveness

Though not our primary aim for this study, our ultimate goal is to assess intervention outcome effectiveness and modify the program as needed to optimize effectiveness. To do this, though, it is necessary to plan in advance for potential issues surrounding the methods of collecting outcome measures [31,45]. Therefore, our second aim was to determine the effectiveness of the VSI specific to the utilization of VA primary care and suicide attempts requiring VA medical care within the first 10 months following military discharge.

##### VA Primary Care Utilization (Primary Outcome)

VA medical record data were obtained from the VA CDW on VA primary care utilization and defined using relevant stop codes (including presence of utilization and dates of service) for all 1102 TSMVs included in analyses. TSMVs who had a VA primary care visit within the first ten months post-military discharge were scored as “yes” for their post-military discharge assessment; otherwise, they were scored as “no”. Some TSMVs were already eligible for VA healthcare and previously sought VA primary care due to meeting the eligibility requirements for VA healthcare because of prior military service. These TSMVs were scored as “yes” for the baseline assessment.

##### Suicide Attempt Requiring VA Medical Care

Baseline data for history of suicide attempt(s) were assessed using the C-SSRS screener [28], completed as part of the military transition application. The C-SSRS screener is a 6-item, self-report scale based on the more comprehensive full-length version of the scale [46]. Previous research suggests the C-SSRS screener has good predictive validity in veteran samples [47]. For the purposes of analyses, TSMVs were considered to have a recent suicide attempt at baseline if they indicated having engaged in a suicide attempt that required medical care within 2 years prior to completing the military transition application.

The presence of a post-military discharge suicide attempt was assessed using VA electronic health record data available through the VA CDW. Similarly to other studies, International Classification of Diseases (ICD)-10 codes T14.91 and Z91.51 were used to identify suicide attempts that resulted in TSMVs requiring VA medical care [48,49]. VA medical records were reviewed for each identified ICD-10 code to confirm that the referenced incident occurred within the TSMVs’ first ten months post-military discharge. These TSMVs were scored as “yes” for their post-military discharge assessment.

### 2.3. Analysis Methods

Primary analyses were done with the R computing environment (R Foundation for Statistical Computing, Vienna, Austria) [50]. As some of the variables had missing values (Table 1), we used multiple imputation (MI) [51]. Following current recommendations on propensity score matching, we used the so-called “within” method where the imputation, matching, and model estimation are done separately within each MI data set, and the results are then combined using Rubin’s rule [52]. We made 20 MI data sets with predictive mean matching by chained equations using the mice package [53]. Missing values for the PHQ-2 and M2C-Q questionnaires were imputed at the item level using polynomial regression to preserve their ordinal character, and sum scores were then computed using the passive imputation approach [53]. Following recommendations from the randomized controlled trial literature [54], the MI data sets were made separately for the VSI and TAU armss to account for any possible treatment-by-covariate interactions.

The minimum sample size for these analyses was determined analytically [55]. With a conventional 80% power level, and at the conventional *α* level of 0.05, the minimum sample size needed was a total of 170 participants (i.e., *n* = 85 per arm) to reliably detect the expected effect size of an odds ratio of 2.4. For matching, we used nearest neighbor matching via the genetic matching algorithm [56] using the MatchThem package (R Foundation for Statistical Computing, Vienna, Austria) [57]. The genetic algorithm optimizes balance among the confounders using the scaled generalized Mahalanobis distance. During the matching procedure, we used the full set of potential confounders listed in Table 1 [57]. Because of the combination of MI and matching, the cases included in each MI data set varied. Therefore, the descriptive statistics reported in Table 1 are averages across the 20 MI data sets, following the procedures recommended by Enders [51]. The total sample size before matching was *n* = 1368. During matching, *n* = 266 TAU cases were dropped in each of the MI data sets, and all *n* = 551 of the VSI cases were retained. Matching balance was assessed with a combination of the statistical and graphic diagnostics outlined in Greifer and colleagues [58]. After matching, the balance was excellent for most of the covariate set.

Separate models were constructed for VA primary care utilization and suicide attempts requiring VA medical care, and we fit the primary substantive models using logistic regression. To protect against imperfect covariate balance, we conditioned the regression models on the full covariate set [59]. To protect against separation in the secondary outcome, we used the bias-correction methods for point estimates and standard errors in the secondary model [60].

For both effectiveness outcomes, we used the average treatment effect on the treated as the causal estimand [59,61], which we computed using the marginal effects package [62]. To account for the dependencies created by the matching, we used cluster robust standard errors to express uncertainty in the estimate. We present the estimands using point-probability contrasts (i.e., risk differences) as the effect-size metric, along with their 95% confidence intervals (CI).

## 3. Results

### 3.1. Aim 1. Evaluate the Feasibility of Adaptations

Adaptation 1. TSMVs Apply Within the Military

All TSMVs enrolled in the study completed the military transition application while serving within the military. The demographics of the TSMVs within the study were like those within the U.S. Army for biological sex. The TSMVs were 82% men and 18% women, which is similar to the proportion of 16% of women who comprise the active-duty U.S. Army [37]. The percentage of enlisted TSMVs (94% vs. 80%) was notably higher in this study than the percentage found within the U.S. Army, with the percentage of officers being lower (6% vs. 20%) [62]. Enrolled TSMVs were also less likely than the broader Army population to be White, non-Hispanic TSMVs (42% vs. 54%).

Adaptation 2. Establish Robust Data Infrastructure

The VA team faced minimal difficulties accessing the data for the TSMVs’ military transition application, VA enrollment and medical records, and VA administrative and outreach data. However, the team did face challenges merging data across these sources so that they could be used for operational and clinical purposes. Although Onward Ops had an advanced data architecture and dedicated staff to manage data from the military transition application, the VA team did not have a comparable architecture nor dedicated staff. Further, the VA team did not have advanced customer relationship management software, which could have organized data across sources and created individual profiles for the TSMVs to facilitate service coordination. Overall, this resulted in inefficiencies and delays in data being available to the VA team.

Adaptation 3. Expand Community Partnerships and Peer Sponsors

Data from the VA CDW identified the post-military destination for 463 of the TSMVs within Arm1/VSI (84%; see online Supplementary Material Table S3). Most TSMVs stayed within region 8 (Texas; 42%), with smaller proportions moving to region 4 (South Carolina, Georgia, Alabama, and Florida; 14%), region 10 (California, Nevada, Arizona, New Mexico, and Hawaii; 12%) or region 5 (Illinois, Indiana, Kentucky, Michigan, Ohio and Tennessee; 10%).

As of April 2023, 21 community partners had entered into agreements with Onward Ops to fulfill local community partner responsibilities. This number expanded to 33 by October 2024, with expansion having already occurred in regions requiring additional sponsors, as identified below [63]. There was variability in the areas covered by each community partner (see online Supplementary Material Table S3). For instance, Onward Ops entered into separate agreements with many partners in New York that operate solely in respective counties whereas Endeavors [64] is the community partner for most of the metropolitan areas within Texas.

By comparing the number of active sponsors and their workload with the projected number of sponsors that would be needed based on the trends identified for the TSMVs in Arm1/VSI, we identified specific regions that will need to increase the number of available sponsors for future VSI efforts (see online Supplementary Material Table S3). We also created heat maps [65] to graphically depict areas within the regions that need additional sponsors (see online Supplementary Material Figure S2). No additional sponsors were needed to initiate the enrollment of TSMVs into the VSI for VSI trial #2 by November 2024. However, a total of 665 new sponsors will be needed to support VSI trial #3 by March 2026.

Adaptation 4. Increase VA Clinical Capacity to Address TSMV Needs

There were key barriers to connecting TSMVs to VA care involving the TSMVs not submitting necessary VA paperwork or not providing sufficient information for VA to make VA eligibility determinations. A total of 450 TSMVs in the VSI arm (82%) submitted a VA registration form; of these, 382 (69% of the total arm) were determined by VA to be eligible for VA clinical services. Additional analysis revealed that 57 ineligible determinations (84%) were related to VA not having enough information from the TSMVs to assess if they met eligibility requirements (e.g., minimum of two years of active-duty service, salary below the minimum threshold, and confirmation of being discharged from the military).

A total of 291 TSMVs (53% of those in the VSI) attended a VA primary care appointment within the data collection period. Of these, 151 TSMVs (27% of all TSMVs in the VSI) were seen within the NVCC and 140 (25%) connected directly with a local VA facility. Most of the TSMVs who attended an NVCC appointment (66%, *n* = 101) received one virtual primary care session before being transferred to a local VA facility in their post-military discharge destination.

The medical conditions most frequently diagnosed by NVCC primary care providers and identified within the VA CDW (see online Supplementary Material Table S4) were related to a musculoskeletal diagnosis (38%, *n* = 57), depression (30%, *n* = 45), anxiety (29%, *n* = 44), post-traumatic stress disorder (22%, *n* = 33), a respiratory diagnosis (17%, *n* = 26) and migraines (15%, *n* = 23). In total, 47% of the TSMVs (*n* = 71) were referred to mental health services and 26% (*n* = 40) were prescribed a psychotropic medication as part of the NVCC appointment.

Women were more likely than men to attend multiple NVCC sessions before connecting with local VA care (48%, *n* = 14 vs. 29%, *n* = 35). Women TSMVs also received mental health referrals (74%, *n* = 23) more often than men (40%, *n* = 48). Relatedly, women TSMVs were also more likely to report military sexual trauma during their NVCC appointment (45%, *n* = 14 vs. 3%, *n* = 3) and more likely to be diagnosed by the providers for anxiety (45%, *n* = 14 vs. 25%, *n* = 30).

### 3.2. Aim 2. Effectiveness

VA Primary Care Utilization (Primary Outcome)

**Figure 2:**
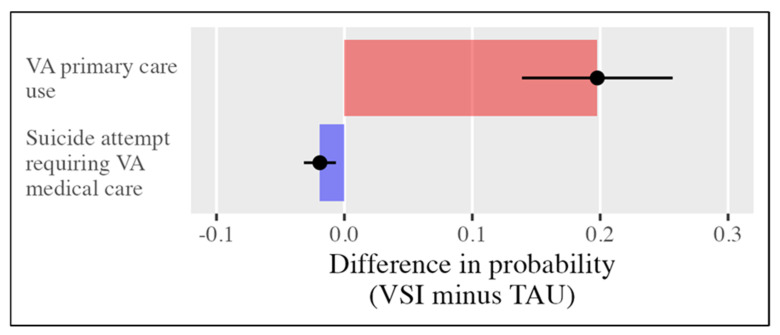
Effectiveness results for primary and secondary outcomes.

We hypothesized that VSI participants would have higher VA primary care utilization than non-VSI matched controls. Across the matched samples in the 20 imputed data sets, the average percentage of VA primary care utilization was 43% overall, 53% for VSI, and 33% for TAU. The point-probability contrast or risk differences from the conditional logistic regression model indicated that the VSI caused a significant increase in VA primary care utilization of 0.198, 95% CI [0.139, 0.257], supporting the research hypothesis for the primary outcome (Figure 2).

Suicide Attempt Requiring VA Medical Care

We hypothesized that VSI participants would also have fewer suicide attempts requiring VA medical care within 10 months of their military discharge, relative to their non-VSI matched controls. Across the matched samples in the 20 imputed data sets, the average percentage of TSMVs with suicide attempts was 0.46% overall, 0.0% for VSI, and 0.92% for TAU. The point-probability contrast or risk differences from the conditional logistic regression model indicated that the VSI caused a significant decrease in suicide attempts of −0.019, 95% CI [−0.032, −0.007], supporting the research hypothesis for the secondary outcome (Figure 2).

## 4. Discussion

This study identified and evaluated the feasibility of four priority adaptations to the VSI after VSI trial #1. Results also highlighted the preliminary effectiveness of the VSI for both the primary (VA primary care utilization) and secondary (suicide attempts requiring VA medical care) outcomes. However, the magnitude of effects for the outcomes differed such that the VSI arm showed a large improvement over TAU for the primary outcome of connecting to VA primary care, whereas it demonstrated only a small improvement over TAU for the secondary outcome of post-military suicide attempts. Though the secondary outcome is a high priority for both our evaluation team and for VA at large, low base rates for suicide attempts among TSMVs limits the ability of any program to make large improvements. It is notable that approximately one percent of the TSMVs in the TAU condition required VA medical care resulting from a suicide attempt within the first ten months post-military discharge, a rate consistent with other studies [26]. These results suggest that the expansion of the core elements of the VSI, consistent with VA’s preventive and public health strategy to suicide prevention, and the adaptations evaluated in this study may help the VSI address the physical and psychological needs of TSMVs and the suicide risk that TSMVs face as they transition from military service to civilian life.

The FRAME helped evaluators to systemically categorize four priority adaptations made to the VSI and identify the action items necessary to increase the feasibility of the VSI prior to initiating subsequent VSI trials. For the first adaptation, the evaluators identified that the percentage of enlisted TSMVs was higher in this study than the percentage found within the U.S. Army. This higher percentage of enlisted TSMVs most likely led to there being less White, non-Hispanic TSMVs in the study than the U.S. Army given that 69% of officers are White, non-Hispanic but only 50% of enlisted soldiers are White, non-Hispanic. In response, in October 2024, U.S. Army staff established procedures to facilitate opportunities for more officers to enroll within the VSI during their out-processing sequence on participating military installations.

Although programs utilizing volunteer peer sponsors may be effective in supporting TSMVs during the transition to civilian life, they require a significant infrastructure to support data management for enrollment/monitoring. Such an infrastructure requires creative solutions and the willingness to coordinate sometimes complex public–private partnerships between stakeholders. Regarding the second adaptation, the evaluators identified that the VA team faced challenges merging the data so that it could be used for operational and clinical purposes. Therefore, in the spring of 2024, the VA team decided to expand the data management personnel assigned to the project and transition the VSI data within the VA system to REDCap [66], a free, secure web-based application that enabled the VA team to collect, merge and better manage the data in a systematic manner.

Specific to the third adaptation, VA evaluators identified that at least 665 new sponsors would be needed for the second trial by March 2026, with special emphasis on regions 4, 5, and 8. It appears that the expansion of the number of community partners within these regions will assist with increasing the number of additional sponsors. In response to the need for new sponsors, Onward Ops and the VA team applied for and received a grant in the fall of 2024 from Face the Fight [67] to recruit and certify the required number of sponsors.

The evaluation of the fourth adaptation revealed that even with the support of trained peer sponsors and VA outreach, nearly half of the TSMVs did not connect with VA primary care in the months following discharge from military service. Without this support, rates of connection were closer to 1 in 3 in the TAU arm. These statistics highlight the significant barriers faced by TSMVs to connect with potentially necessary medical services, providing further evidence of a “deadly gap” as most veterans who die by suicide are not connected to VA care [40]. Certain barriers were related to the TSMVs not submitting necessary paperwork or not providing necessary information, as 18% of the TSMVs in Arm1/VSI did not submit a VA registration form and 13% submitted the VA registration form but were determined to be ineligible due to the VA not having enough information to make eligibility determinations. In response, the VA team produced and published a video in December 2024 [68] that provides step-by-step instructions for TSMVs to successfully complete the form and to educate them on the necessary information needed by the VA. The VA team now sends the video link to the TSMVs as part of their outreach activities.

TSMVs connecting with VA primary care presented a range of medical concerns, emphasizing the need for efficient connection to a range of specialty care post-military discharge. Offering virtual primary care may help increase the accessibility of these services for about half of the TSMVs who eventually connect and may be particularly engaging for women TSMVs. Women TSMVs were more likely to disclose military sexual trauma and anxiety-related difficulties through these appointments, suggesting the need for facilitating efficient connection to needed mental health care. To better understand the unique challenges faced by women TSMVs and to improve the quality and type of VA healthcare services for them within subsequent VSI trials, the NVCC established a partnership with the VA Women’s Health Research Network [69]. In October 2024, in response to the high prevalence of musculoskeletal and mental health diagnoses, the NVCC also established a virtual physical therapy capability and expanded the resources available for mental health services.

The results of the current study should be understood within the context of a few limitations. First, although the quasi-experimental design provided opportunities to compare a group of TSMVs who received VSI support with a comparison, the absence of randomization and inclusion of TSMVs who declined services increased the likelihood that unassessed confounding factors not integrated as part of the matching process may have influenced the results. Second, the evaluation only considered VA primary care use and post-military discharge suicide attempts that were identified within VA medical records, which potentially led to the impact of the VSI being underestimated as the TSMVs within the TAU arm were less likely to utilize VA healthcare and have a VA medical record. For subsequent VSI trials, evaluation will also include the administration of the C-SSRS at follow-up time points after military discharge. This will ensure a more consistent measurement of suicide attempts in future study groups and address a portion of this limitation. Third, VSI studies only enroll active-duty servicemembers from the U.S. Army, thus making it unclear how the VSI would impact servicemembers from other military branches.

## 5. Conclusions

Our study evaluated and attempted to increase the feasibility of the adaptations made to the VSI after the original VSI trial. The results also add to a growing body of research on VSI effectiveness, specifically highlighting effects on access to VA primary care services and suicide attempts after military discharge. The findings broadly support the continued implementation and evaluation of the VSI as an intervention to assist TSMVs through the deadly military-to-civilian transition gap. The VSI also helps VA in meeting requirements to improve the support of TSMVs, as outlined within the Commander John Scott Hannon Veterans Mental Health Care Improvement Act.

We are not aware of any other evaluations assessing the impact of an intervention specific to the studied outcomes that initiates the enrollment of TSMVs while they are still in the military. Such evaluation is necessary for federal agencies to meet requirements under the Foundations for Evidence-Based Policymaking Act (U.S. Public Law 115–435) of 2018, which requires agencies to publish annual evaluation plans that demonstrate the use of evidence and evaluation to inform policies and budget allocations [70]. Of note, the VSI is integrated within VA’s plans [71] for fiscal years 2024, 2025, and 2026 as a suicide prevention program. Therefore, the current evaluation has important implications for informing the national implementation of interventions for TSMVs.

## Figures and Tables

**Table 1:** Characteristics of transitioning servicemembers/veterans in the matched effectiveness analyses.

Variable (Categorical)	Category	Overall (*n* = 1102) ^a^			VSI (*n* = 551)			TAU (*n* = 551)			
		*n*	%	%_miss_	*n*	%	%_miss_	*n*	%	%_miss_	Δ%
Gender	Women	204	18.51	0.21	105	19.11	0.18	99	17.91	0.25	−1.20
	Men	898	81.49	0.21	446	80.89	0.18	452	82.09	0.25	
Race ^b^	American Indian or Alaska Native	46	4.18	11.8	23	4.24	14.16	23	4.12	9.45	0.12
	Asian	84	7.60	11.8	46	8.26	14.16	38	6.93	9.45	1.32
	Black or African American	306	27.80	11.8	157	28.45	14.16	150	27.16	9.45	1.29
	Native Hawaiian or Other Pacific Islander	38	3.42	11.8	21	3.77	14.16	17	3.07	9.45	0.70
	White	701	63.63	11.8	352	63.85	14.16	349	63.40	9.45	0.44
Rank	Enlisted	1034	93.79	0.71	514	93.32	1.27	519	94.26	0.15	0.94
	Officer	68	6.21	0.71	37	6.68	1.27	32	5.74	0.15	
Ethnicity	Non-Hispanic	798	72.44	3.14	398	72.26	5.08	400	72.62	1.19	0.36
	Hispanic	304	27.56	3.14	153	27.74	5.08	151	27.38	1.19	
Prior VA Registration	Yes	75	6.81	0.00	38	6.90	0.00	37	6.72	0.00	0.17
	No	1027	93.19	0.00	513	93.10	0.00	514	93.28	0.00	
Prior VA Enrollment	Yes	16	1.43	0.00	8	1.45	0.00	8	1.41	0.00	0.05
	No	1086	98.57	0.00	543	98.55	0.00	543	98.59	0.00	
Prior VA Primary Care Visit	Yes	4	0.36	0.00	2	0.36	0.00	2	0.36	0.00	0.00
	No	1098	99.64	0.00	549	99.64	0.00	549	99.64	0.00	
Suicide Attempt 2 Years Prior	Yes	18	1.63	0.00	13	2.36	0.00	5	0.91	0.00	1.45
	No	1084	98.37	0.00	538	97.64	0.00	546	99.09	0.00	
**Variable (Continuous)**		***M***	***SD***	**%_miss_**	***M***	***SD***	**%_miss_**	***M***	***SD***	**%_miss_**	***SMD***
Age (Years)		26.64	4.99	0.09	26.39	4.78	0.18	26.89	5.18	0.00	−0.10
Military to Civilian Questionnaire		24.62	12.19	4.91	25.05	12.55	3.99	24.19	11.80	5.83	0.07
Patient Health Questionnaire-2		3.10	1.66	25.57	3.16	1.69	17.97	3.03	1.62	33.18	0.08
STARRS Risk Score [27]		0.01	0.02	31.24	0.01	0.02	45.55	0.01	0.02	16.93	0.10

Note. VSI = Veteran Sponsorship Initiative; TAU = Transition as Usual; %_miss_ = Percent of values missing; Δ% = The point percentage difference by group; SMD = The standardized mean difference (aka Cohen’s d) between arms. Because of the combination of multiple imputation and matching, the cases included in each MI data set varied. Therefore, the descriptive statistics reported are averages across the 20 MI data sets, following the procedures recommended by Enders [50]. ^a^ The total sample size before matching was N = 1368. During matching, *n* = 266 TAU cases were dropped in each of the MI datasets, and all *n* = 551 of the VSI cases were retained. ^b^ Race categories were not mutually exclusive.

## Data Availability

Data available through contacting corresponding author.

## Supplementary Materials

The following supporting information can be downloaded at https://www.mdpi.com/article/10.3390/ijerph23040519/s1; Table S1: VA Veteran Sponsorship Initiative Studies Timeline and Components. Table S2. VSI Framework for Reporting Adaptations and Modifications-Expanded. Table S3. Percent of TSMVs from Arm1/VSI Moving to Regions, Community Partners per Region and Number of Sponsors Needed for two Additional VSI Trials. Table S4. VA National Virtual Care Clinic for Transitioning Veteran Primary Diagnoses and Mental Health Procedures. Figure S1. Consolidated Standards of Reporting Trials Diagram. Figures S2. Regional Heat Maps with Overlap of Projected TSMVs for two VA studies vs. current certified Sponsors.

## Abbreviations

The following abbreviations are used in this manuscript:

CDW	VA Corporate Data Warehouse
CI	Confidence Intervals
CRADA	Cooperative Research and Development Agreement
C-SSRS	Columbia-Suicide Severity Rating Scale
DoW	Department of War
ETS	Expiration Term of Service
FRAME	Framework for Reporting Adaptations and Modifications-Enhanced
ICD	International Classification of Diseases
M2C-Q	Military to Civilian Questionnaire
MI	Multiple Imputation
NVCC	VA National Virtual Care Clinic for Transitioning Veterans
PHQ-2	Patient Health Questionnaire-2
SMART	Specific-Measurable-Achievable-Relevant-Timely
SMD	Standardized Mean Difference
STARRS	DoW Study to Assess Risk and Resilience in Servicemembers
TAU	Transition As Usual
TSMV	Transitioning Servicemember/Veteran
US	United States of America
VA	United States Department of Veterans Affairs
VISN	Veterans Integrated Service Network
VSI	VA Veteran Sponsorship Initiative
