# Supplementary material for: Mitigating Suicide Risk During the Military-to-Civilian Transition: The VA Veteran Sponsorship Initiative
Source: Int J Environ Res Public Health. 2026 Apr 17;23(4):519. doi: 10.3390/ijerph23040519 (PMC13116221; doi:10.3390/ijerph23040519)
Supplement: Supplementary file 1 [file ijerph-23-00519-s001.zip › ijerph-4191990-supplementary.pdf]

International Journal of
Environmental Research and Public Health

MDPI

Supplementary Materials

Table S1. *VA Veteran Sponsorship Initiative Studies Timeline and Components*

This table captures the different VSI studies that have already been completed, as well as funded future studies.

			2022				2023				2024				2025					
	Type Study	2021	Jan-Mar	Apr-Jun	Jul-Sep	Oct-Dec	Jan-Mar	Apr-Jun	Jul-Sep	Oct-Dec	Jan-Mar	Apr-Jun	Jul-Sep	Oct-Dec	Jan-Mar	Apr-Jun	Jul-Sep	Oct-Dec	2026	2027
**VSI Trial #1 (2014-2018, n=200)**																				
Arm 1: Waitlist Control Condition (n=65)	Randomized Controlled Trial																			
Arm 2: Community-based Veteran Service Organization Only (n= 67)																				
Arm 3: Community-based Veteran Service Organization And Onward Ops sponsor (n=68)																				
**Current Study (2021-2024, n=1,102)**																				
Priority adaptations made to VSI program (Apr 2021-Apr 2023)	Longitudinal, Quasi-Experimental and Matched-Cohort Pilot Study	Priority adaptations made to VSI program																		
Arm 1: Transition As Usual (Matched Control Condition; n=551)								Enrollment window on military installations (Apr 2023- Dec 2023)			Transition to Civilian Life Window									
Arm 2: Onward Ops sponsor & facilitated connection to VA clinical services (n=551)																				
**VSI Trial #2 (2022-2025, n=630)**																				
Arm 1: Transition As Usual (Control Condition; n=315)	Randomized Hybrid type 2 Effectiveness-Implementation Trial: Phased enrollment for arms per military installation		Enrollment window on military installations (Feb 2022- Dec 2023)																	
Arm 2: Onward Ops sponsor & facilitated connection to VA clinical services (n=315)														Enrollment window on military installations (Nov 2024 -Dec 2025)						
**VSI Trial #3 (2025-2027, n=2,700)**																				
Arm 1: Top 30% of predicted risk; Transition As Usual (Control Condition; n=900)	Randomized Hybrid type 2 Effectiveness-Implementation Trial: Simultaneous enrollment for arms within phased roll-out to military installations																		Enrollment window on military installations (2026 to 2027)	
Arm 2: Top 30% of predicted risk; Onward Ops Only (n=900)																				
Arm 3: Top 30% of predicted risk; Onward Ops sponsor & facilitated connection to VA clinical services (n=900)																				

Table S2. *VSI Framework for Reporting Adaptations and Modifications-Expanded*
This table captures the application of the Frame-work-for-Reporting-Adaptations-and-Modifications-Expanded (FRAME) to systematically categorize adaptations made to VSI.

**Adaptation**	***1. TSMVs Apply within the Military***	***2. Establish Data Infrastructure***	***3. Increase Community Partnerships and Peer Sponsors***	***4. Increase VA Clinical Capacity to Address TSMV Needs***
FRAME Construct / Feasibility Dimension	**Recruitment Capability**	**Data Collection Procedures**	**Integration Into Existing Systems**	**Practicality**
**WHEN did modification occur? *(All occurred prior to Aim 2)***	October 2022	April 2021 to April 2023	April 2021 to April 2023	April 2021 to April 2023
**Were adaptations PLANNED?**	Planned/Proactive	Planned/Proactive	Planned/Proactive	Planned/Proactive
**WHO made the decision to modify?**	U.S. Army leadership	VA Innovation Ecosystem; Onward Ops	VA HAP; Onward Ops	VA Undersecretary for Health; VA Office of Primary Care; VA NVCC
**WHAT was modified?**	Contextual/ Population	Scale Up Activities	Scale Up Activities	Content: Adding Elements
**At what LEVEL OF DELIVERY (for whom/what is the modification made?)**	Target Intervention Group	Organization	Organization	Cohort
**What was the goal?**	Improve Effectiveness: Enroll all TSMVs prior to military discharge	Increase feasibility: Seamless flow of data from OO to VA	Increase Reach: Solidify roles & responsibilities	Rapid access to virtual PC & specialty care
**Reasons**	Organization Mission: Enroll TSMVs prior to entering the deadly gap	Organization Mission. Coordinate care for TSMVs	Organization Mission: VA community suicide prevention strategy	Organization Mission: Seamless access to VA healthcare

Note. TSMV= Transitioning Servicemember/Veteran; NVCC= VA National Virtual Care Clinic for Transitioning Veterans; VA HAP= National Center for Healthcare Advancement & Partnerships

Table S3. *Percent of TSMVs from Arm1/VSI Moving to Regions, Community Partners per Region and Number of Sponsors Needed for two Additional VSI Trials*
This table captures the VSI regions and the number of community partners, TSMVs, and sponsors for each region, as well as the number of sponsors needed for future studies.

Region	U.S. States	Community Partners (As of April 1, 2023)	Community Partners (As of October 1, 2024)	TSMVs Moving to Region from Arm 1/VSI	% of TSMVs/ Region	Number of New Sponsors Needed for VSI trial #2 by November 2024	Number of New Sponsors Needed for VSI trial #3 by March 2026
1	ME, RI, NH, VT, CT, MA	1	1	3	0.65%	0	0
2	NY, PA, NJ, DE	13	15	21	4.54%	0	0
3	WV, VA, NC	0	0	29	6.26%	0	0
4	SC, GA, AL, FL	0	3	66	14.25%	0	97
5	TN, KY, MI, OH, IN	1	2	48	10.37%	0	67
6	LA, AR, MS	0	0	8	1.73%	0	10
7	IL, MO, KS, WI, MN, NE, IA, ND, SD	0	1	13	2.81%	0	0
8	TX	1	3	193	41.68%	0	491
9	WA, OR, UT, MT, WY, ID, UT, CO, OK	2	5	28	6.05%	0	0
10	CA, NV, AZ, NM	3	3	54	11.66%	0	0
	Total	21	33	463		0	665

Table S4. *VA National Virtual Care Clinic for Transitioning Veteran Primary Diagnoses and Mental Health Procedures*

This table describes the outcomes for each of the participants who received treatment from the virtual clinic during the study period.

	Total (*n* = 151)		Men (*n* = 120)		Women (*n* = 31)		
	n	%	*n*	%	*n*	%	Δ%
**Musculoskeletal Diagnoses**							
Any	57	37.75	43	35.83	14	45.16	9.33
Upper Extremities (Shoulder/Neck/Wrist/ Back)	46	30.46	35	29.17	11	36.67	6.32
Lower Extremities (Hip, Knee, Ankles, Feet)	30	19.87	21	17.50	9	29.03	11.53
**Mental Health Diagnoses**							
Depression	45	29.80	33	27.50	12	38.71	11.21
Anxiety	44	29.14	30	25.00	14	45.16	20.16
PTSD	33	21.85	26	21.67	7	22.58	0.91
Alcohol substance use disorder	21	13.91	18	15.00	3	9.68	-5.32
**Other Diagnoses**							
Respiratory disease: Chronic rhinitis/Allergic rhinitis/Asthma/COPD/allergies (other)	26	17.22	21	17.50	5	16.13	-1.37
Migraines (including patients with documented migraines with treatment during time in service/Tinnitus)	23	15.23	15	12.50	8	25.81	13.31
Gastrointestinal	21	13.91	15	12.50	6	19.35	6.85
Military sexual trauma	17	11.26	3	2.50	14	45.16	42.66
Obstructive sleep apnea	16	10.60	13	10.83	3	9.68	-1.16
Elevated blood pressure (with or without diagnosis of Hypertension)	12	7.95	11	9.17	1	3.23	-5.94
Traumatic brain injury	6	3.97	6	5.00	0	0.00	-5.00
**Mental Health Procedures**							
Psychotropic medication prescription	40	26.49	29	24.17	11	35.48	11.32
Mental health referral	71	47.02	48	40.00	23	74.19	34.19

Note. Δ% = The point percentage difference of women minus men, for which positive values indicate higher percentages for women, and negative values mean the reverse.

Figure S1. *Consolidated Standards of Reporting Trials Diagram*

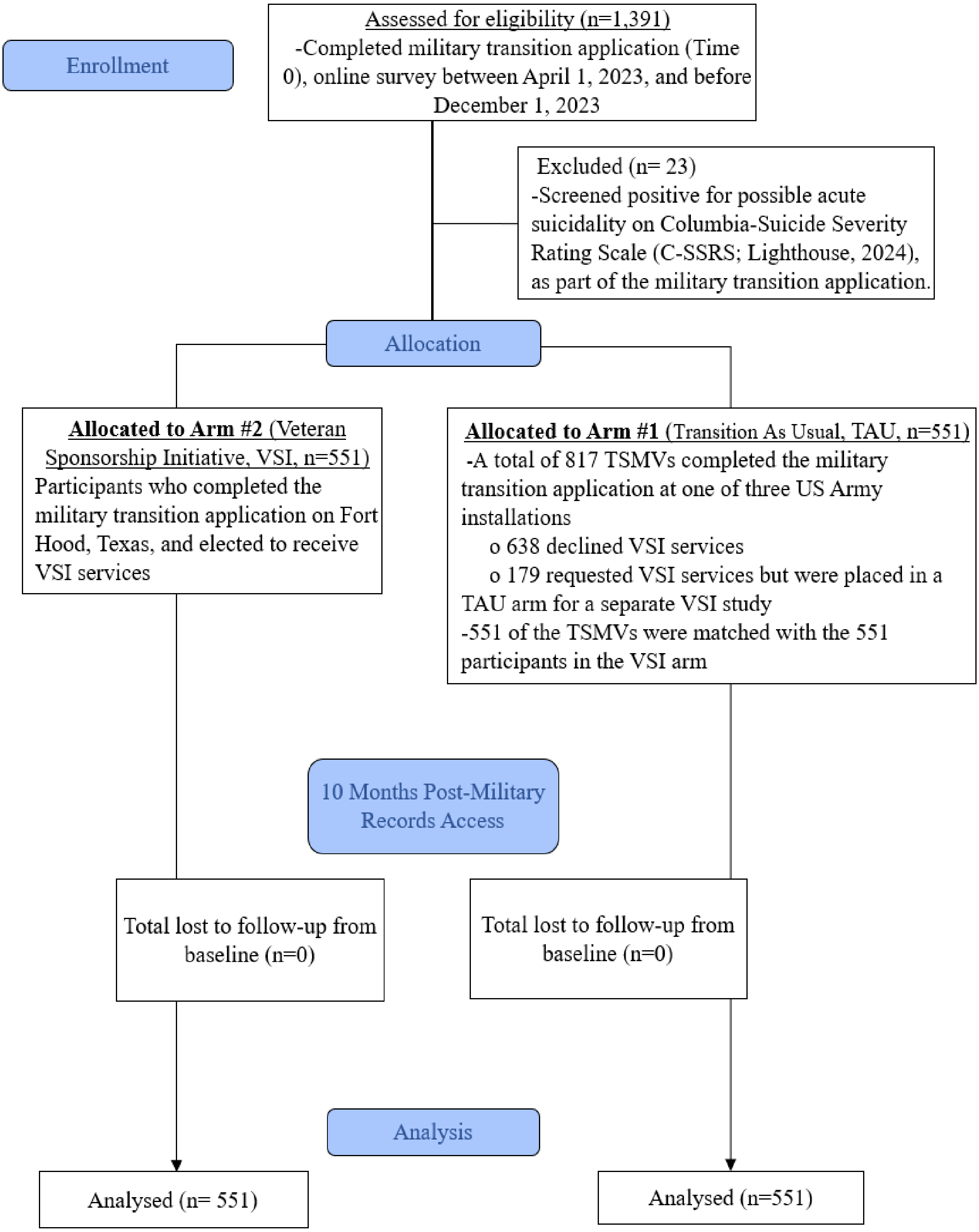

Figures S2. *Regional Heat Maps with Overlap of Projected TSMVs for two VA studies vs. current certified Sponsors*

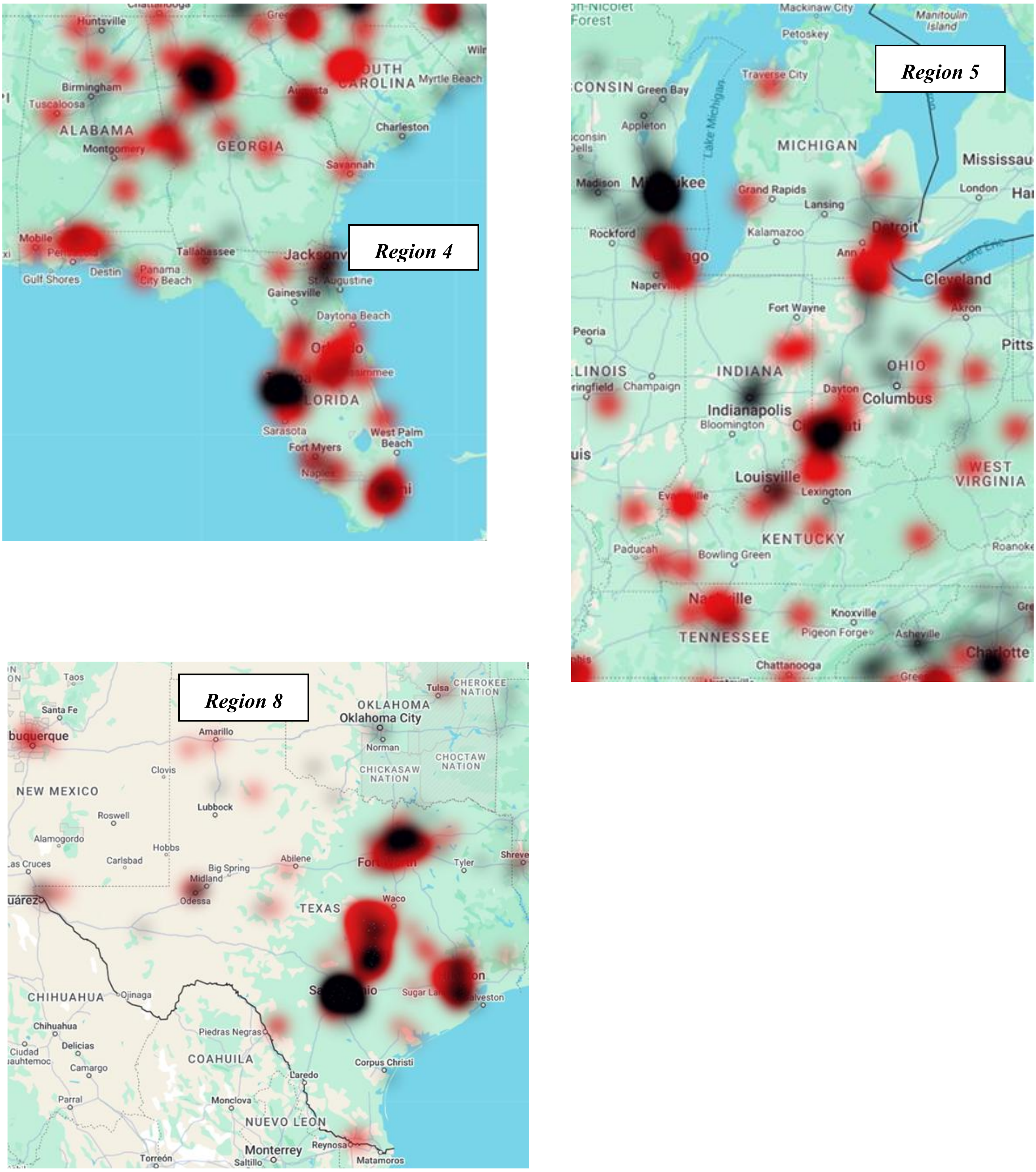

Note.

Red=Projected Transitioning Servicemembers/Veterans (TSMVs)

Black=Projected Sponsors

The more prominent color identifies an imbalance of projected TSMVs vs. certified Sponsors. The red areas identify areas with more projected TSMVs compared to active sponsors. The black areas identify areas with more current/active sponsors compared to projected TSMVs.
